# Issues in applications of nursing care robots, and in the training of care workers in their use in Japan

**DOI:** 10.3389/fmed.2025.1459015

**Published:** 2025-04-30

**Authors:** Nghia Chi Nguyen, Masami Saito

**Affiliations:** ^1^Faculty of Management and Law, Aomori Chuo Gakuin University, Aomori, Japan; ^2^Graduate School of Economics and Management, Tohoku University, Sendai, Japan; ^3^Department of Early Childhood Education, Aomori Chuo Junior College, Aomori, Japan

**Keywords:** nursing care robots, disadvantages, roles of caregivers, education, benefits

## Abstract

**Introduction:**

Although Information and Communication Technology (ICT) care robots are being used to address the shortage of human resources in nursing care in Japan, there are few studies on the application and disadvantages of robotics in caring for older persons.

**Methods:**

The authors surveyed seven students taking a nursing care course based on a self-administered questionnaire after they experienced various ICT nursing care robots in the education program. The responses were coded and analyzed qualitatively to consider the advantages and disadvantages of using robots in nursing care and the roles of nursing care workers.

**Results:**

While nursing care robots have been evaluated for their contributions to caring for older persons, they also have disadvantages, such as high cost, discomfort, and risk of injury.

**Conclusion:**

Education programs for welfare students should include active experience with robots and a deep understanding of their advantages and disadvantages in caring for older people. Further collaboration between development companies, training schools, and care sites is essential to help educators and students get used to nursing care robots soon, refer to their experience in testing these robots, and simultaneously study potential disadvantages and discomforts of nursing care robots.

## 1 Background

### 1.1 Current status and predictions of Japan’s aging population

As of October 1, 2019, Japan’s total population was 126.17 million people, and the proportion of people aged 65 and over in the total population was increasing. The proportion of these older adults was 28.4%. In 1950, it was less than 5%, in 1970, it rose to 7%, and in 1994, it exceeded 14%. The population is now estimated to fall below 120 million in 2029, 100 million in 2053, and 88.08 million in 2065. In addition, the number of people over 65, the baby boomer generation who supported Japan’s postwar economic development, reached 33.87 million in 2015. By 2025, the baby boomer generation over 75 years of age will consist of an estimated 36.77 million people (1).

In this way, Japan’s aging rate continues to rise, and by 2036, those over age 65 will reach 33.3%, or one in three older adults. It will continue to rise after that; by 2065, it will reach 38.4%, or 1 in 2.6 people. In 2065, the proportion of adults aged 75 years or older will reach 25.5% or approximately 1 in 3.9 people. Among older people, those aged 65–74 years are referred to as the early older persons, and those aged 75 years and older are considered the late older persons. The problem with these increasing rates is that as the population gets older, the demand for health care increases due to declines in physical, mental, and social functions. The peak number of early older adults (65–74 years old) was 17.68 million in 2016, and the number of late older persons (over 75 years old) is expected to continue to increase until 2054 (1).

The average number of workers from younger generations supporting each older adult was 12.1 in 1950 but is predicted to be only 1.3 by 2065.

### 1.2 Extension of average lifespan

Compared to some other major countries in the world (Italy, Switzerland, France, Canada, Germany, the United Kingdom, and the United States), the data from 2016 showed that the average life expectancy for women and men in Japan was the highest. Furthermore, the average life expectancy in Japan was nearly 60 years for men and over 62 years for women in 1950, but by 2000, 50 years later, it had increased to over 77 years for men and nearly 85 years for women. The average life expectancy continues to increase, by 2065, this figure is expected to reach 84.95 years for men and 91.35 years for women (2). Along with the increase in average life expectancy has arisen the challenge of more and more people becoming bedridden for long periods due to health problems. The country must work on solutions to ameliorate this situation.

### 1.3 Current status and trends of people requiring care

The number of people certified to require nursing care support in Japan increased by approximately 2.90 times over the 17 years from the end of April 2000 (when nursing care insurance first became available) to April 2016.

The classification of nursing care support required consists of seven levels, starting with the mildest condition: support required 1 to 2, plus nursing care required 1 to 5 (Table 1).

**Table 1:** Level of care required and types of support.

Level of care required	Standard time for nursing care certification	Types of Support
Support required 1	25–32 min	Direct living assistance: Support with bathing, excretion, meals, etc. Indirect living assistance: Support with housework such as laundry and cleaning Problem, behavior-related behaviors: Search for wandering, clean up after unclean behavior, etc. Functional training-related activities: Functional training such as walking and daily living training. Medical-related procedures: Assist with medical care such as administering infusions and treating bedsores.
Support required 2	32–50 min	
Nursing care required 1	32–50 min	
Nursing care required 2	50–70 min	
Nursing care required 3	70–90 min	
Nursing care required 4	90–110 min	
Nursing care required 5	Over 110 min	

Source: Ministry of Health, Labour and Welfare (https://www.mhlw.go.jp/topics/kaigo/kentou/15kourei/sankou3.html).

The number of people certified as having mild conditions has increased the most. This rate has been reported to be increasing still. Additionally, long-term nursing care insurance differ based on the proportion of older adults in each municipality. The national average premium for 3 years was JPY 2,911 per month in 2000, but in 2018, it rose to JPY 5, 869 per month. Furthermore, the average charge is predicted to increase to JPY 9,200 by 2040 (3).

### 1.4 Securing nursing care personnel

#### 1.4.1 Current status and outlook of nursing care workers

The status of care workers is highly dependent on non-regular employment, with 61% of care workers working in nursing care facilities being regular workers, and 39% being non-regular workers. Furthermore, among visiting caregivers, 30.3% were regular employees and 69.7% were non-regular employees. Looking at nursing care workers by gender, the ratio of women is high; while most men working in nursing care are under 40 years of age, most women are over 40 years of age. Although the number of registered care workers increased by 875,262 from 2000 to 2012; currently, only approximately half of the registered population is engaged in nursing care employment (4).

After the implementation of the long-term care insurance system, the number of people certified as requiring long-term care increased, and the number of care workers increased from 2000 (549,000) to 2012 (1,532,000) due to the increase in service fees they can receive and to the aforementioned needs of the aging population. This number has increased by approximately three times over the past 12 years. As the number of older adults increases, it is predicted that 2.37–2.49 million nursing care workers will be required as soon as 2025.

#### 1.4.2 Factors that hinder securing care workers

While nursing care jobs have a positive image among the public, there are concerns about difficult work, night shifts, and salary. It has been pointed out that the image of a job with a low salary or with uncertainty about the future impedes the recruitment of nursing care workers (4).

Preparing and improving the workplace environment are discussed as one of four initiatives to secure nursing care personnel in “Opinions regarding the review of the nursing care insurance system.” Specifically, the development of nursing care robots should be promoted to reduce the burden on nursing care staff, and the development and improvement of the workplace environment should be conducted to improve communication and work efficiency using ICT (5).

Existing studies mainly examined artificial intelligence and robotics in the health ecosystems (6), norms and values in using them (7), nursing students’ technological literacy (8), the benefits in adopting robotics in clinical practice (9, 10), especially in surgery, patient rehabilitation, medication storage, and contacting patients or colleagues in other countries outside Japan (11).

However, there is hardly any study on the application of robotics in nursing care and education programs for care workers in Japan.

## 2 Literature review

### 2.1 Definition of a nursing care robot

**Figure 1:**
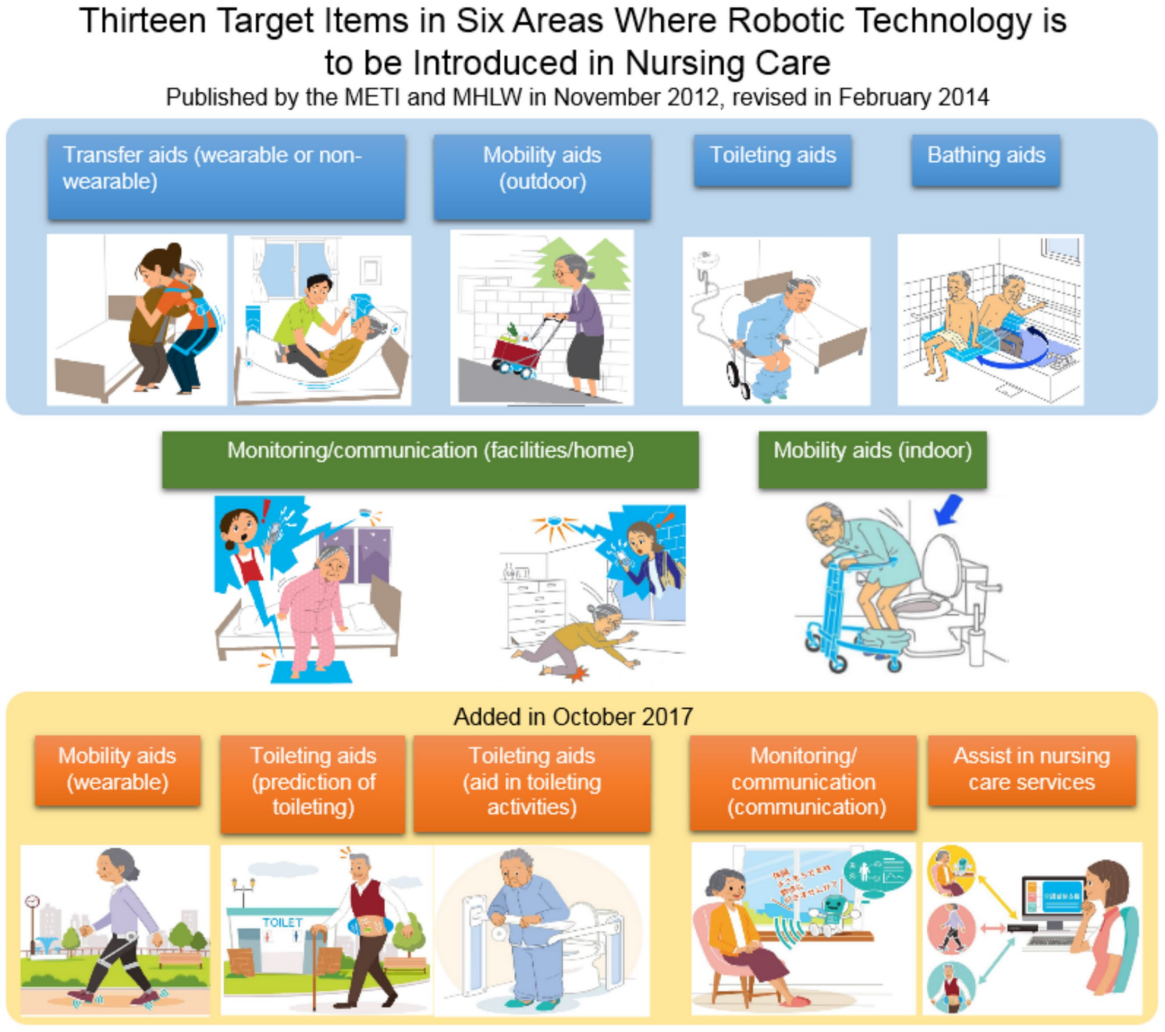
Priority areas for nursing care robots. Source: Japan Agency for Medical Research and Development (https://www.amed.go.jp/en/program/list/12/02/001.html),

The definition of a robot as set by the Ministry of Health, Labor, and Welfare is an intelligent mechanized system with elemental technology that senses information (sensor systems), (2) makes judgments (intelligence/control systems), and (3) operates (drive systems). Nursing care robots can be applied as devices that support user independence and reduce the burden on caregivers. Regarding the development of robotic nursing care equipment, a joint study committee between the Ministry of Economy, Trade and Industry and the Ministry of Health, Labor and Welfare was established in June 2012, and in 2013 five items (transfer aids—wearable or non-wearable, mobility aids—outdoor, toileting aids, and monitoring at facilities) were announced as priority areas. In February 2014, areas of mobility aids—indoor, monitoring at home, and bathing aids were added to create eight items in five areas (Figure 1). Furthermore, in October 2017, this number expanded to 13 items in six areas.

### 2.2 Purpose of utilizing ICT nursing care robots

As a challenge for nursing care, it is predicted that over the 15 years from 2010 to 2025, the aging rate (people reaching the age of 65 and over) is significantly increasing, from 23 to 30% of total population. It has become clear that the baby boomer generation will become older persons more or less all at once, and that the number of nursing care workers required will be 2.5 million by 2025. Additionally, given the current situation in which 70% of nursing care workers report that they suffer from lower back pain, it is necessary to reduce their physical burdens. The Ministry of Health, Labor and Welfare surveyed 220 nursing care facility managers and nursing care staff to determine the burden of each type of nursing care (the types shown in Figure 1) (12). Among the nursing care issues, The Ministry of Economy, Trade and Industry has designated “priority areas” that need to be addressed using nursing care robots.

To develop these robot care devices, equipment development will be led by the Ministry of Economy, Trade, and Industry, and private companies and research institutes will utilize advanced technology to provide support for older adults and nursing care facilities. Support for the development of equipment was provided based on the specific needs of nursing sites. In addition, the Ministry of Health, Labor, and Welfare plays a role in conducting demonstrations at nursing care sites from the early stages of development by communicating on local needs and by monitoring and evaluating prototype devices at nursing care sites.

### 2.3 Existing studies

To understand how research on ICT nursing robot education has been conducted to date, a Google Scholar search in Japanese was conducted using the keywords “ICT nursing robot education” and “care worker training.” Of the 609 results, the most popular papers centered on care worker education are listed in Table 2.

**Table 2:** Prior research on ICT education on nursing care robots.

Study	Results and research points
Yoshikawa (15)	In nursing care worker training classes, nursing care robots and ICT, introduced in classroom lectures, can be connected to learning with real experience “by touching and doing them.” Future developments, such as ICT recording nursing care, are expected.
Kumita (16)	In Japan, care using robots and the like has yet to become available daily. It is mentioned that welfare robots are lent to training schools by companies in Denmark, and the students are learning nursing care techniques using the latest technology.
Yokoyama et al. (17)	A questionnaire survey was conducted at 311 nursing care facilities to clarify the introduction and utilization status of nursing care support equipment and the educational status of nursing care staff. As a result, the adoption rate was found to be 52.1% for sliding boards and 2.7% for wearable transfer support suits. 63.1% lacked sufficient manuals, pointing out the need for more uniform education within the facilities.
Tomikawa (18)	As a result of conducting a questionnaire survey on the understanding of no-lifting care in 401 nursing care worker training courses nationwide, it was pointed out that training instructors themselves often teach behavior that ignores worker safety and comfort, contributing to the shortage of nursing care personnel. It is mentioned that there is an urgent need for improvement of knowledge and skills of nursing care trainers, clarification of policies regarding occupational health, and no-lifting care education. Government, nursing care educators, and academic organizations need to support these efforts.
Asaishi (19)	Pointing out that peripheral work is not included in the 2019 “Nursing Care Site Innovation Plan,” it is necessary to actively introduce welfare equipment to nursing care peripheral work and to discuss the work hierarchy to be mechanized. It is mentioned that it is necessary to deepen the definition of peripheral work and to accumulate evidence of the effectiveness of specific welfare equipment in the future.
Kakutani et al. (20)	As a result of clarifying the significance and issues of using communication robots for four third-year high school students studying welfare, Kakutani et al. (20) raised the necessary elements to acquire practical nursing care skills that combine technology and engineering, unique to STEAM (Science, Technology, Engineering, Arts, Mathematics) education. The conclusion is that robotics fosters the ability to learn and helps develops caregivers’ humanity.

A similar methodology was used to search for English literature on Google Scholar. A literature review of studies published between 2010 and 2019 on the application of robotics in nursing in the Cochrane Library, ScienceDirect, and ProQuest databases yielded over 400 hits, but most were not directly related to nursing and only 26 studies were chosen because of their relevance to the topic (11). The results showed that robotics are often used in surgery, in patient rehabilitation, in medication storage, and in making contact with patients or colleagues. As new technologies, robotics are gradually playing more important roles in nursing. The review of McAllister et al. (11) emphasized the importance of nurse training that encourages the creative use of robotics in nursing without compromising the relationship between nurses and patients.

However, there is hardly any study on the application of robotics in nursing care and education programs for careworkers when using robotics in nursing care settings. In the context of nursing care in Japan, the authors conducted a literature review titled “ICT nursing care robot education” and “nursing care worker training” to understand how research on such ICT education has been conducted thus far in Japan (Table 2).

## 3 Research method

### 3.1 Data collection method

There is a problem with the number of students attending nursing care schools in Japan. As of April 2024, there were 54 schools offering 4-year programs, 4 schools offering 3-year programs, 211 schools offering 2-year programs, and 12 schools offering 1-year programs on nursing care in Japan. College A of our research offers a 1-year program, which is rare nationwide. In addition, a decrease in the number of students entering the program has been a nationwide issue recently, with three training schools in the fieldwork Prefecture suspending their recruitment. The number of students entering the welfare course at the target school has been low, with only three in 2022, seven in 2023, and five in 2024 out of a capacity of 25. Furthermore, this decrease results from the fact that people can work in the nursing care field without qualifications, and there is a negative image that it is tough and has a low salary compared to other occupations.

Although the advantages of nursing care robots are explained in care worker training textbooks, it is essential to confirm these advantages and examine potential disadvantages. Data from students’ experiences aiming to become care workers has never been investigated. The participants were seven students taking a nursing care course at College A to obtain certification as care workers. In ICT nursing care robot programs 3 and 4, students experienced using the various ICT nursing care robots listed in Table 3 in an off-campus exhibition hall. The participants then completed a self-administered questionnaire.

**Table 3:** Nursing care robots students used, partial list.

Type	Product name Company Name	Purpose	Unit cost (Is the nursing care insurance rental applicable?)
Communication support	Voice recognition communication robot Chapit RayTron, INC.	A voice recognition communication robot that is resistant to noise and has a high word-recognition rate. It is capable of entirely hands-free and smooth conversation, can understand over 500 words, and can memorize 200 monthly schedules.	\135,000 (excluding tax) (August 2016) Insurance not currently applicable
Monitoring/communication support	Watching over the nursing care Aams Bio Silver Co., Ltd.	This mat-shaped monitoring support robot can monitor heartbeat, breathing, body movements, landing on the floor, sleeping status, and more, from a remote location. It can monitor and support users 24 h a day.	\178,000 (excluding tax) Insurance not currently applicable
Mobility assistance	ROBOHELPER SASUKE Muscle, Inc.	Transfer from bed to wheelchair. Suitable for people with a height of 140 to 180 cm and up to 120 kg.	\998,000 (October 2019) Insurance not currently applicable
Mobility assistance	Transfer support robot HugLT1-02 FUJI Corporation.	Transfer between sitting positions such as from bed to wheelchair and from wheelchair to bathroom, and maintaining standing position for putting on and taking off clothing. Supports up to 100 kg	\980,000 (April 2018) Nursing care insurance welfare equipment Applicable for rental
Mobility support	Robot Assist Walker RT.2 RT.WORKS Co., Ltd.	The automatic electric assist function detects road location and road conditions, enabling safe and comfortable walking.	\118,000 (excluding tax) Welfare equipment rental is covered by nursing care insurance
Excretion support	Wrappon Aile 2 Nihon Safety Co., Ltd.	Automatically seals excrement and odors using thermo-compression bonding without water, reducing the burden of cleaning receptacles	\89,800 or \128,000 yen Insurance not currently applicable
Excretion support	Dfee Professional Triple W Japan Inc.	An ultrasonic sensor displays and informs users of the level of urine accumulation in an easy-to-understand 10-step scale.	\300,000 *Dedicated Pad sold separately 3,980 yen (5-disc set) April 2022 Certified as specific welfare equipment certification

Source: Aomori Welfare Plaza Exhibition Robot List, 2023.

### 3.2 Analysis method

In the 3rd and 4th classes of the ICT nursing care robot program listed in Table 4, students spent 90 min experiencing the nursing care robots listed in Table 3 in the nursing care robot exhibition room at the Welfare Plaza while receiving an explanation about them. These robots are not available in College A’s training rooms. A self-administered questionnaire for target participants was carried out after the 3^rd^ and 4^th^ classes of the ICT nursing care robot program listed in Table 4.

**Table 4:** ICT care robot education curriculum.

Session number	Content	Method	Venue	Outline
1	What is a nursing robot?	Lecture	On campus	Study the definition, purposes, background, and trends of nursing care robots in Japan.
2	Current status, issues, and examples of nursing care robots	Lecture	On campus	Study how ICT nursing care robots are being used and the challenges applying them in nursing care settings (remaining difficult though they are being incorporated into nursing/welfare education). In addition, we explain that information about nursing care robots is necessary to be gathered on-site at nursing care facilities.
3	Nursing care robot experience	Practice	Off-campus exhibition hall	During fieldwork at Welfare Plaza, students will receive an explanation of the ICT care robot and observe/use it for 90 min.
4	Nursing care robot experience	Practice	Off-campus exhibition hall	
5	Advantages and disadvantages of nursing care robots	Practice	On campus	Reflect on the experience with ICT nursing robots and consider the advantages and disadvantages of using them.
6	Advantages and disadvantages of nursing care robots	Practice	On campus	
7	Advantages and disadvantages of nursing care robots	Practice	On campus	
8	Nursing care robot summary	Practice	On campus	Each student prepares material for a 7-min presentation using PowerPoint.
9	Nursing care robot summary	Practice	On campus	
10	Students from another university attend. Presentations, Q&A, summary of the course.	Practice	On campus	Present in 7 min and follow with 2 min Q&A. By implementing inter-university class collaboration and asking and answering questions from the perspective of students studying in fields other than nursing care and welfare, students can gain a multifaceted learning experience. Those asking these questions will also have the opportunity to learn about the challenges faced in nursing care as Japan ages.

The authors surveyed seven students who observed and experienced using the robots. The responses were analyzed using qualitative data analysis methods and text mining. The responses on the benefits of nursing care robots, disadvantages, and roles of care workers while using nursing care robots in supporting and caring for users were organized and classified into categories, subcategories and codes (Tables 5–7). Consequently, 11 categories, 22 subcategories, and 38 codes were identified. The authors designate [Category], *Subcategory*, and <Code>.

**Table 5:** List of benefits after the use of nursing care robots *n* = 7.

Category	Subcategory	Code	Representative data
Improve operational efficiency	Reduce burden	Reduced travel distance	Travel distance is reduced
	Expected to improve operational efficiency	Work becomes smoother	Collaborate with other staff regardless of distance (2)
			Hands-free, and does not interfere with work (2)
			Data can be entered immediately after assistance
Easy independence support	Lower back pain prevention	Lower back pain prevention	Lower back pain prevention
		Reduced physical burden	No need for power (3)
			Easy to move, even for women (5)
			Light
	Independence support	Possibility of independence support	You can easily go where you want to go by yourself
		Individual care	It definitely takes time
Stress reduction	Stress reduction	Not touching filth	You can dispose of waste without touching it (9)
		Freeing up time	Frees up time for other support
		No smell	No smell (6)
	Easy	It’s easy	Easy (3)
			No need to worry about processing (2)
			Isn’t it convenient for being at home?
Safe environment	Can be observed from a distance	Fall prevention	Fall prevention
			The device can be adjusted to the user’s condition (2)
		Can be observed from a distance	You can see the user’s situation in detail (3)
			Understand the situation from a distance (3)
	Health care	Accurate health observation can be done automatically	Get accurate vital numbers
			Recorded automatically
Reducing dementia progression	Healing	Eliminate loneliness	Possibility of eliminating loneliness (3)
			Cuddling
		Healing	Healing
	Dementia reduction	Promote conversation	Can have a conversation (2)
			Suppressing the progression of dementia

**Table 6:** List of disadvantages after the use of nursing care robots *n* = 7.

Category	Subcategory	Code	Representative data
Feeling burdened by high costs	Feeling of burden	Feeling of cost burden	Feeling of burden for users due to high costs (10)
Risk of unusableness	Risk of accidents	Possible machine error	Depending on the situation, you may not be able to understand what is being said (4)
		Risk of accident	Accidents can occur with cords (3)
	Mechanical malfunction	Processing takes time	It takes time to process
		Network instability	Network instability is dangerous (2)
		Requires charging	Requires charging (7)
Physical and mental burden on both users and caregivers	Feeling of burden of assistance	Feel burdensome when putting on and taking off	Difficult to put on and take off (7)
		Difficult to move	It seems difficult to move (3)
		Feel uncomfortable	loud mechanical noise
	Mental burden	Privacy cannot be protected	User privacy may not be protected
		Feel anxious	Are not users worried because there is no human voice or presence?
		feel a sense of shame	Does not it make you feel ashamed?
Dangerous environment	Difficult to understand health status	Unable to check excrement	May not be able to grasp the shape
	Wired fall risk	Risk of wired hazards	Isn’t a wired device dangerous?
			Complicated to install
Inconvenience of use	Difficulty in use	Compatible with only specified words	I feel lonely because I can speak only a certain number of words.
		Inaccuracy in hearing	I cannot understand the dialect (4)
			Inaccurate hearing (4)
	Need space	Need a large space	Requires large space

**Table 7:** Role of care worker using ICT care robots *n* = 7.

Category	Subcategory	Code	Representative data
Provide support that is close to users	Increase engagement with users and provide support	Support that is close to users	Support that is more sensitive to the user’s feelings can be provided
			Can focus on psychological support
		Increase engagement with users	Reducing the burden on users (2)
			Able to interact with users and do other things
Improving awareness at nursing care sites and mastering the use of nursing care robots	Ability to master nursing care robots	It is important to master nursing care robots	Master many types of nursing care robots
	Reforming awareness in nursing care settings	Reforming awareness in nursing care settings	Improving the awareness of nursing staff
		Introduction of new knowledge	Understand the incorporation of new knowledge

### 3.3 Nursing care robot program

The content of the care worker training curriculum to be conducted in 10 lessons (90 min each) on life support techniques was as follows (Table 4).

## 4 Results and analysis

### 4.1 Benefits of nursing care robots

As a result of the analysis, two subcategories and two codes were extracted from the category [Improve operational efficiency]. *Reduced burden* (reduction in travel distance) and *Expected to improve operational efficiency* were answered by <Reduced travel distance> and <work will become smoother>.

Four codes were extracted from two subcategories from [Easy independence support], <lower back pain prevention> and <physical burden reduction> from *Lower back pain prevention*, and <possibility of independence support> and <individual care> from *Independence support* were extracted.

Two subcategories and four codes were extracted from [Stress Reduction]. For *stress reduction*, the responses were <not touching filth>, <freeing up time*>*, and <no smell*>*. For *Easy*, the response was <it’s easy>.

Two subcategories and three codes were extracted from [Safe Environment]. *Can be observed from a distance* means <fall prevention> and <can be observed from a distance>. And *health care* means <accurate health observation can be done automatically>.

Two subcategories and three codes were extracted from [reducing dementia progression]. *Healing* means <Eliminate loneliness> and <healing>. *Alleviating dementia* means <promoting conversation>.

### 4.2 Disadvantages of using the nursing-care robots

The results regarding the disadvantages in using the nursing-care robots are shown in Table 6. [Feeling burdened by high costs] has one subcategory and one code, from *Feeling of burden* to <Feeling of burden due to costs>.

Two subcategories and five codes were extracted based on [Risk of unusableness] category. For *Risk of accidents* subcategory, the responses were <Possible machine error> and <Risk of accident>. The answers for the subcategory *Mechanical malfunction* were <Processing takes time>, <Network instability> and <Requires charging>.

Two categories and six codes were extracted from [Physical and mental burden on users and caregivers]. Regarding the subcategory *Feeling of the burden of assistance*, the responses were <Feeling burdensome when putting on and taking off>, <Difficult to move>, and <Feeling uncomfortable>. Regarding *Mental burden* subcategory, the responses were < Privacy cannot be protected >, <Feeling anxious> and <Feeling shame>.

There are two subcategories and two codes for [Dangerous environment]. For the subcategory *Difficult to understand health status*, the answer was <Unable to check excrement>; and *Wired fall risk* subcategory had the correlate <Risk of wired hazards>.

Two subcategories and three codes were extracted based on the [Inconvenience of use] category. In terms of *Difficulty in use*, it was <Only compatible with specific words> and <inaccuracy in listening>. For *Need space*, the response was <Need a large space>.

#### 4.2.1 Role of care workers in using ICT nursing-care robots

The results for the roles of care workers using ICT care robots are shown in Table 7. One subcategory and two codes were extracted from [Provide support close to users]. In the subcategory *Increase engagement with users and provide support,* the correlates were <Support that is close to users> and <Increase engagement with users>.

## 5 Discussion

The stated advantages of using robots after the student subjects experienced them are considered to be within expectations; however, their mention of the disadvantage of high user cost was interesting. Costs need to be lowered to make robotic devices easier for everyone to obtain. Although devices may reduce the physical and mental burdens on both caregivers and users, high costs may be a hindrance to their introduction.

Regarding the cost of care robots, for example, the transfer-care type HAL costs 2 million yen per unit, and the transfer-care type robot-assisted walker RT.2 costs 118,000 yen. Although it is necessary for education, it is difficult to introduce them because of their high cost.^1^ Care robots are advanced devices and systems that support the lives of elderly people who need care and contribute to reducing the burden on caregivers and their families. They are already widespread in welfare-advanced countries such as Denmark and the Netherlands, and their usefulness is being proven. As shown in Table 4, the average price of a care robot is about 400,000 yen, making it expensive, and in a report from a research project on the effectiveness of care robots conducted by the Ministry of Health, Labour and Welfare, the most common issue felt when introducing care robots was high introduction costs (13). In addition, concerning the risk of injury, Mitsubishi Research Institute, Inc., with funding from the Ministry of Health, Labour and Welfare, presented a collection of points to use safely based on actual near misses regarding the use of care robots in 600 facilities in March 2021 (14).

Furthermore, in some cases, using the product may pose a risk of injury or cause discomfort, and care workers need to consider not only the benefits but also the disadvantages of nursing care robots. This awareness will promote the introduction of safer ICT nursing care robots.

## 6 Conclusion

Although they are included in nursing care worker education, nursing care robots are expensive, so in real terms their introduction has been limited. Many in the field remain reluctant to introduce robots in nursing care settings. This research was carried out in a nursing care education program where students had a lot of hands-on experiences, and they thought deeply about the benefits and disadvantages for caregivers and users. It was verified that the role of care workers is to master and use ICT care robots to provide close support to users, which can improve overall awareness in care settings.

As the introduction of care robots in the field has not progressed, there are few opportunities for care worker training schools to use care robots in their training sites. Since there are limits to introducing ICT nursing care robots as training equipment in nursing care worker schools, it is necessary to offer opportunities for active experience using public institutions such as those detailed in this program. It is essential to focus on both the advantages and disadvantages of ICT care robots and to consider their ongoing role. The authors believe developing human resources that understand nursing care robots in the field is necessary. These findings will improve the quality of nursing care by being used in future nursing care settings.

The Ministry of Health, Labour and Welfare has reported on its achievements from 2016 to 2019, in which development companies and care sites have discussed the direction of care robot development from the idea stage before the development of the robot and proposed development proposals.^2^ It is believed that the quality of care robot education can be ensured if teachers and students at care worker training schools can also participate at this stage. For example, in Japan, the Ministry of Economy, Trade and Industry has been promoting the development of such robots, so we recommend that further collaboration with nursing care educators be included in the development requirements to help educators and students get used to nursing care robots soon, refer to their experience in testing these robots and study potential disadvantages and discomforts of nursing care robots at the same time.

## Data Availability

The datasets presented in this study can be found in online repositories. The names of the repository/repositories and accession number(s) can be found in the article/supplementary material.

## Footnotes

^1^ https://www.nippku.com/kaigo-seisansei/care-robot/

^2^ https://www.mhlw.go.jp/stf/seisakunitsuite/bunya/0000209634.html
